# Low-to-Moderate Dosage and Short-Term Use of Corticosteroids Benefit Patients With Severe COVID-19 Infections

**DOI:** 10.3389/fmicb.2022.953328

**Published:** 2022-07-19

**Authors:** Liuqing Yang, Ling Peng, Weibo Wu, Mengli Cao, Chuming Chen, Fuxiang Wang, Jennifer St. Sauver, Yingxia Liu

**Affiliations:** ^1^Shenzhen Third People’s Hospital, Shenzhen, China; ^2^Robert D. and Patricia E. Kern Center for the Science of Health Care Delivery, Mayo Clinic, Jacksonville, FL, United States

**Keywords:** Coronavirus Disease 2019 (COVID-19), corticosteroid, propensity score matching (PSM), mortality rate, severe and critical illness

## Abstract

Although the FDA has given emergency use authorization (EUA) for some antiviral drugs for the treatment of COVID-19, no direct antiviral drugs have been identified for the treatment of critically ill patients, the most important treatment is suppression of the hyperinflammation. The purpose of this study was to evaluate the role of corticosteroids in hospitalized severe or critical patients positive for COVID-19. This is a retrospective single-center descriptive study. Patients classified as having severe or critical COVID-19 infections with acute respiratory dysfunction syndrome in Shenzhen Third People’s Hospital were enrolled from January 11th to March 30th, 2020. Ninety patients were classified as having severe or critical COVID-19 infections. The patients were treated with methylprednisolone with a low-to-moderate dosage and short duration. The days from the symptom onset to methylprednisolone were about 8 days. Eighteen patients were treated with invasive ventilation and intensive care unit (ICU) care. All the patients in the severe group and ten in the critical group recovered and were discharged. Three critical cases with invasive ventilation died. Although cases were much more severe in the corticosteroid-treated group, the mortality was not significantly increased. Early use of low-to-moderate dosage and short duration of corticosteroid may be the more accurate immune-modulatory treatment and brings more benefits to severe patients with COVID-19.

## Introduction

Since the outbreak in December 2019 of Coronavirus Disease 2019 (COVID-19) in Wuhan, China, the disease has spread rapidly to other areas. Till May 13, 2022, the COVID-19 pandemic, as the largest public health crisis in a century, resulted in approximately 517.6 million cases and 6.26 million deaths (World Health Organization [WHO], 2022). Mild cases of COVID-19 can be treated without hospitalization, while severe COVID-19 can cause excessive inflammation in a short period of time, leading to acute respiratory distress syndrome (ARDS) and even death. The pros and cons of glucocorticoids in the treatment of COVID-19 have been controversial (Angus et al., 2020; Dequin et al., 2020; Fadel et al., 2020; Lu et al., 2020; Russell et al., 2020; Wu et al., 2020a,b; Group et al., 2021; Jeronimo et al., 2021). The research team has rich experience in the treatment of severe viral pneumonia (H7N9, H5N1, and H5N6); we believe that corticosteroid treatment is a double-edged sword. We are more concerned about how to use the advantages of glucocorticoids and reduce side effects, thereby minimizing patient mortality and shortening ICU time (Shang et al., 2020). Overall, there is a need for more clinical data to support that corticosteroid treatment does more benefit than harm to patients with COVID-19. A multi-center randomized controlled trial (the RECOVERY study) from Oxford University that gets a lot of attention was never allowed by the ethics committee to be implemented in China, because it included patients who did not receive oxygen or oxygen only, and such mild patients were not indications of glucocorticoid therapy in China from many years ago, and the actual study results supported the experience of China (Group et al., 2021). Thus, the objective of this study is to describe the clinical characteristics and outcomes of patients hospitalized with severe COVID-19 and treated with corticosteroids.

## Materials and Methods

### Study Oversight

This is a retrospective, single-center study. We recruited patients from January 11th to March 30th, 2020, at Shenzhen Third People’s Hospital, China (Shenzhen Third People’s Hospital is the only hospital designated by the government for COVID-19 treatment in Shenzhen, and all confirmed cases of COVID-19 found in the region were sent to this hospital). Oral consent was obtained from all the patients. This case series was approved by the institutional ethics board of Shenzhen Third People’s Hospital. All the patients with COVID-19 were diagnosed according to the World Health Organization interim guidance (World Health Organization [WHO], 2020). Ninety patients classified as having severe or critical COVID-19 infections were enrolled from January 11th to March 30th, 2020. Clinical outcomes (i.e., discharges, death) were monitored, and the last date of follow-up was March 30th.

### Disease Classification

Laboratory confirmation of COVID-19 was performed in two different institutions: the Chinese CDC and Shenzhen Key Laboratory of Pathogen and Immunity (Shenzhen Third People’s Hospital). Throat-swab specimens from the upper respiratory tract or sputum specimens were obtained from all the patients at admission. Cases were diagnosed using quantitative reverse transcriptase-polymerase chain reaction (qRT-PCR) (GeneoDX Biotechnology Co., Ltd.). Other respiratory viruses, including influenza A virus, influenza B virus, respiratory syncytial virus, Adenoviridae, Mycoplasma, and Chlamydia, were also examined by real-time RT-PCR.

After admission, sputum or endotracheal aspirates were obtained every other day for identification of possible causative bacteria or fungi. Additionally, all the patients underwent chest X-ray or CT.

All confirmed cases were then clinically classified according to the Chinese guideline “pneumonia diagnosis and the treatment program for new coronavirus infection (Trial Version 6),” as follows: 1, mild – with fever, respiratory symptoms, and radiographic manifestations of pneumonia; 2, severe – meet any of the following: (a) respiratory distress, respiratory rate (RR) ≥30 times/min; in resting state, refers to oxygen saturation ≤93%; partial arterial oxygen pressure (PaO_2_)/oxygen absorption concentration (FiO_2_) ≤300 mmHg (1 mmHg = 0.133 kPa); 3, critical – meeting one of the following conditions: (a) respiratory failure, and the need for mechanical (invasive) ventilation; (b) shock; (c) complicated with other organ failure requiring intensive care.

### Data Sources

The research team of the Department of Infectious Disease, The Third People’s Hospital of Shenzhen updated data forms daily from electronic medical records (EMRs) with regard to epidemiological, clinical, laboratory, and radiological characteristics and treatment and outcomes. The data were reviewed by two trained physicians (WW and LP).

We describe signs and symptoms on admission, comorbidities, laboratory results, treatment received for COVID-19, and clinical outcomes (discharged or death).

### Statistical Analysis

The statistical analysis in this study was performed by R v3.6.2 Propensity Score Matching (PSM) for age, sex, smoking history, and comorbidity was used to generate matched cohorts for comparison. MatchIt (Ho et al., 2011) version 3.0.2 was used to perform PSM, with an optimal matching method and a case/control ratio of 1:2 or 1:3. The number of standard deviations of distance measurements in which the control unit (caliper) was plotted was set to 0.02. We performed PSM two times on the ninety cases. First, to compare the mortality of corticosteroid- and corticosteroid-free patients, twenty-seven patients were included in the no-corticosteroid group and fifty-four in the corticosteroid group. We next performed a *t*-test and Fisher’s exact test for continuous variables (e.g., age) and categorical variables (e.g., gender, smoking history, comorbidity) to test for differences between these two groups of cohorts.

Then, to compare the therapeutic effects of severe (non-invasive ventilation) and critical (invasive ventilation) patients, we performed PSM again using the original ninety patients, with eighteen cases (the critical group) and fifty-four controls (the severe group), comprising 80% (72/90) of the patients in this study. We then performed a *t*-test and Fisher’s exact test for continuous variables (e.g., age) and categorical variables (e.g., sex, smoking history, comorbidity) to assess differences between these two groups. The significance level was set to 5% in this study (*p* < 0.05, two-sided). Continuous variables were compared with the Mann-Whitney *U* test, and the results are expressed as the median [interquartile ranges (IQR)]; categorical variables were compared by the χ^2^ test or Fisher’s exact test between severe (no ICU care) and critical (ICU care) groups, and the results are expressed as the number (%).

Multivariable logistic regression analysis was performed to examine the independent association of corticosteroid therapy on mortality with independent variables, including corticosteroid therapy age, smoking history, comorbidities, WBC, lymphocyte count, CRP, PCT, CD4+ cell count, PaO_2_/FiO_2_, immunoglobulin therapy, antibiotic treatment, and days from the onset to admission.

A two-sided α of less than 0.05 was considered statistically significant. The statistical analyses were performed using SAS software, version 9.4, unless otherwise indicated.

## Results

Ninety patients with confirmed severe COVID-19 were enrolled from January 11th to March 30th, 2020. Clinical outcomes (i.e., discharge, death) were monitored, and the last date of follow-up was March 30th. Eighteen patients were treated with invasive ventilation and intensive care unit (ICU) care. All hospitalized patients in our study received antiviral treatment with lopinave/litonawe (LPV/r) or ribavirin and interferon on admission. We used propensity score matching (PSM) to identify individuals with similar characteristics to serve as our cohorts.

Based on the first PSM, twenty-seven patients with COVID-19 were included in the corticosteroid-free group and fifty-four in the corticosteroid group. There was no significant difference in mortality between the groups. All patients in the critical group who received invasive ventilation were treated with methylprednisolone (MP). BiPAP (bilevel positive airway pressure) ventilation and high-flow nasal catheter oxygen (HFNCO) were more common in those administered corticosteroids (Table 1). CD4+ and CD8+ cell counts in the corticosteroid treatment group were significantly lower than those in the corticosteroid-free group (Table 2). Although cases were more severe in the corticosteroid treatment group, there was no significant increase in mortality.

**TABLE 1:** Outcomes in patients with and without corticosteroid.

Outcomes	No. (%) total (*N* = 81)	Corticosteroid-treated group (*n* = 54)	Corticosteroid – free (*n* = 27)	*P*-value
Died	3 (3.7)	3 (5.6)	0	0.5472
Critical (invasive ventilation)	17 (21.0)	17 (31.5)	0	0.0004
Severe (non-invasive ventilation)	64 (79.0)	37 (68.5)	27 (100)	0.0433
Nasal catheter or face mask for oxygen therapy	31 (48.4)	13 (35.1)	18 (66.7)	–
HFNC	17 (26.6)	12 (32.4)	5 (18.5)	–
BiPAP ventilation	16 (25)	12 (32.4)	4 (14.8)	–
Secondary infection	11 (13.6)	10 (18.5)	1 (3.7)	0.0895
Antibiotics treatment	40 (49.4)	29 (53.7)	11 (40.7)	0.3474

*HFNC, high flow nasal cannula; BiPAP, bilevel positive airway pressure.*

**TABLE 2:** Laboratory results on admission of patients with COVID-19.

Laboratory indexes	Normal range	Median (IQR) total (*N* = 81)	Corticosteroid-treated group (n = 54)	Corticosteroid – free (*n* = 27)	*P*-value
White cell count (×10^9^/L)	3.5–9.5	4.64 (3.84–6.27)	4.40 (3.68–6.5)	4.74 (3.83–5.85)	0.8092
Lymphocyte count (×10^9^/L)	1.1–3.2	1 (0.83–1.34)	0.99 (0.76–1.30)	1.07 (0.83–1.44)	0.1560
Platelet count (×10^9^/L)	125–350	156 (127–186)	145 (123–177)	172 (126–189)	0.0675
Alanine aminotransferase (U/L)	9–40	26.2 (19–41)	26.7 (19–42)	26 (19–30)	0.3784
Aspartate aminotransferase (U/L)	15–40	33 (26–43)	35 (25–43)	29 (26–43)	0.3480
Albumin (ALB, g/L)	40–55	40.3 (37.8–43.4)	39.2 (37–42.6)	42.1 (37.7–44.7)	0.0145
Globulin (GLB, g/L)	20–40	25.3 (24.2–29.8)	25.6 (23.9–30.4)	25.1 (24.2–26.9)	0.8107
Creatinine, μmol/L	57–111	72 (57–93)	72 (59–93)	65 (57–89)	0.5333
Creatine kinase (U/L)	50–310	69 (53–126)	73 (54–122)	66 (54–135)	0.9948
Creatine kinase-MB (μg/L)	<0.22	0.89 (0.5–1.27)	0.84 (0.39–1.41)	0.86 (0.52–1.09)	0.9369
D-dimer (mg/L)	0–1.5	0.47 (0.36–0.71)	0.53 (0.37–0.74)	0.4 (0.32–0.67)	0.1180
Cholinesterase (CHE)	5,000–12,000	7,103 (6,250–8,193)	7,047 (5,792–7498)	8,019 (6,237–8,791)	0.0269
Lactate dehydrogenase (U/L)	120–250	268 (205–461)	310 (205–567)	241 (205–429)	0.5004
PCT (Procalcitonin, ng/mL)	<0.1	0.06 (0.05–0.08)	0.06 (0.05–0.09)	0.052 (0.03–0.07)	0.0892
IL-6 (pg/mL)	0–7	23.7 (14.9–39.5)	29.4 (15.6–46.2)	23.3 (16.2–25.9)	0.0784
CD4+ cell count (/μL)	400–500	320 (208–483)	258 (183–391)	348 (204–561)	0.0069
CD8+ cell count (/μL)		169 (108–293)	144 (95–218)	314 (119–500)	0.0028
CRP (C-reactive protein, mg/L)	<8	25.7 (11–52.4)	26.3 (16.6–55.3)	25.1 (12.3–49)	0.5727
PaO_2_/FiO_2_	400–500	348 (281–403)	342 (272–378)	361 (339–423)	0.0501
Chest X-ray findings on admission-*n* (%)					1.0000
Unilateral pneumonia		3 (3.7)	2 (3.7)	1 (3.7)	–
Bilateral pneumonia		78 (96.3)	52 (96.3)	26 (96.3)	–

*Data are median (IQR, interquartile range) or n/N (%), where N is the total number of patients with available data. P-values comparing corticosteroid treated and corticosteroid-free groups are from χ^2^, the Fisher’s exact test, or Mann–Whitney U test.*

Then, to compare the therapeutic effects of severe (non-invasive ventilation) and critical (invasive ventilation) cases, we performed PSM again using the original ninety patients, with eighteen cases in the critical group and fifty-four in the severe group. All the patients in the severe group and ten in the critical group recovered and were discharged. Five critical cases with invasive ventilation remained hospitalized, and three of these patients died. The demographics, signs and symptoms on admission, laboratory results, and treatment conditions are shown in Tables 3–5. Most of the laboratory test results (routine blood examination, liver or kidney function, etc.) and clinical manifestations were not significantly different in the severe or critical group initially after admission.

**TABLE 3:** Demographics and baseline characteristics of 68 patients with COVID-19.

Characteristics	No. (%) total (*N* = 72)	Severe (*n* = 54)	Critical (invasive ventilation) (*n* = 18)	*P*-value
Age, median (IQR), years	62 (56–67)	61 (55–66)	65 (60–68)	0.492
BMI (Body Mass Index)	24.2 (22.2–26.6)	23.9 (22.4–26.6)	24.4 (22.1–26.8)	0.9317
Sex				0.571
Female	32 (36.1)	21 (38.9)	5 (27.8)	
Male	44 (61.1)	33 (61.1)	13 (72.2)	
Ever been to Wuhan or Hubei				1
Yes	54 (75)	40 (74.1)	14 (77.8)	
No	18 (25)	14 (25.9)	4 (22.2)	
Smoking history				0.307
Yes	6 (8.3)	1 (1.9)	2 (11.1)	–
No	66 (91.7)	53 (92.6)	16 (88.9)	–
**Comorbidities**				
No comorbidities	24 (33.3)	20 (33.3)	4 (24)	0.387
Cardiovascular disease	28 (38.9)	20 (37.0)	8 (44.4)	0.780
Diabetes	11 (15.2)	7 (13.0)	4 (22.2)	0.570
Chronic lung disease	5 (6.9)	3 (5.6)	2 (11.1)	0.789
Chronic kidney disease	0	0	0	NA
Chronic liver disease	0	0	0	NA
Hematological disease	0	0	0	NA
**Signs and symptoms on admission**				
Fever	62 (86.1)	46 (85.2)	16 (88.9)	1
Cough	43 (59.7)	32 (59.3)	11 (61.1)	1
Dyspnea	5 (6.9)	3 (5.6)	5 (29.4)	0.0201
Gastrointestinal symptoms	4 (5.6)	2 (3.7)	2 (11.1)	0.2586
No symptoms	4 (5.6)	3 (5.6)	1 (5.6)	1
Days from onset to admission	5 (3–7)	5 (3–7)	5 (3–6)	0.718

*Data are median (IQR, interquartile range) or n/N (%), where N is the total number of patients with available data. P-values comparing severe and critical groups are from χ^2^, the Fisher’s exact test, or Mann–Whitney U test.*

**TABLE 4:** Laboratory results on admission of patients with COVID-19.

Laboratory indexes	Normal range	Median (IQR) total (*N* = 72)	Severe (*n* = 54)	Critical (*n* = 18)	*P*-value
White cell count (×10^9^/L)	3.5–9.5	4.85 (3.84–6.28)	4.68 (3.81–5.69)	6.21 (4.33–6.98)	0.0215
Lymphocyte count (×10^9^/L)	1.1–3.2	1 (0.81–1.27)	1.05 (0.82–1.28)	0.96 (0.81–1.19)	0.5641
Platelet count (×10^9^/L)	125–350	152 (125–185)	154 (127–189)	148 (118–178)	0.7555
Alanine aminotransferase (U/L)	9–40	26 (19–41)	26 (20–44)	23 (16–34)	0.3564
Aspartate aminotransferase (U/L)	15–40	33.6 (26–43)	33 (26–43)	34.3 (23–45)	0.9629
Albumin (ALB, g/L)	40–55	39.8 (37.5–43)	41.75 (38.4–43.2)	35.3 (34.6–39.7)	0.0012
Globulin (GLB, g/L)	20–40	25.4 (23.7–28.5)	25.3 (24.1–27.8)	25.6 (22.6–29.5)	0.8773
Creatinine, μmol/L	57–111	69.8 (57–97)	72 (57–98)	65 (55–77)	0.5357
Creatine kinase (U/L)	50–310	69 (54–132)	81 (52–107)	118 (63–424)	0.1761
Creatine kinase-MB (μg/L)	<0.22	0.95 (0.71–1.55)	0.77 (0.73–1.42)	1.15 (0.69–1.875)	0.1383
D-dimer (mg/L)	0–1.5	0.54 (0.36–0.955)	0.47 (0.3–0.76)	0.97 (0.49–1.71)	0.0121
Cholinesterase (CHE)	5,000–12,000	7,047 (5,877–8,019)	7,131 (6,519–8,431)	5,877 (4,941–6,936)	0.0027
Lactate dehydrogenase (U/L)	120–250	310 (206–555)	307 (205–461)	380 (225–624)	0.2515
PCT (Procalcitonin, ng/mL)	<0.1	0.064 (0.047–0.084)	0.061 (0.035–0.074)	0.077 (0.063–0.131)	0.1607
IL-6 (pg/mL)	0–7	23.7 (14.9–43.1)	23.4 (14.4–33.3)	42.98 (28.2–54.4)	0.0253
CD4+ cell count (/μL)	400–500	271 (209–391)	258 (212–407)	281 (172–370)	0.543
CD8+ cell count (/μL)		144 (106–221)	169 (112–273)	137 (81–179)	0.1381
CRP (C-reactive protein, mg/L)	<8	32 (12.5–54.0)	27.7 (10.5–52.4)	38.6 (22.6–72.3)	0.0378
PaO_2_/FiO_2_	400–500	343 (269–403)	349 (306–413)	310 (194–354)	0.0419
Chest X-ray findings on admission-*n* (%)					0.2646
Unilateral pneumonia		4 (5.6)	2 (3.7)	2 (11.1)	–
Bilateral pneumonia		67 (93.1)	51 (94.4)	16 (88.9)	–

*Data are median (IQR, interquartile range) or n/N (%), where N is the total number of patients with available data. P-values comparing severe and critical groups are from χ^2^, the Fisher’s exact test, or Mann–Whitney U test.*

**TABLE 5:** Treatment and outcomes of patients with COVID-19.

Treatment and outcomes	No. (%) total (*N* = 72)	Severe (*n* = 54)	Critical (*n* = 18)	*P*-value
Corticosteroid-median (IQR)	57 (79.2)	39 (72.2)	18 (100)	0.0154
Dosage of MP (mg/kg/d)	0.73 (0.63–0.89)	0.74 (0.65–0.89)	0.73 (0.62–0.86)	0.8249
Cumulative dosage of MP (mg)	240 (160–300)	230 (160–300)	240 (180–300)	0.8714
Duration of MP treatment (days)	5 (3–5)	4 (3–5)	5 (4–5)	0.5991
Days from onset to MP	8 (6–11)	9 (6–11)	7 (6–9)	0.2593
Antibiotic treatment	41 (56.9)	25 (46.3)	16 (88.9)	0.002
Antifungal treatment	9 (12.5)	3 (5.6)	6 (33.3)	0.006
Intravenous immunoglobulin therapy	55 (76.3)	39 (72.2)	16 (88.9)	0.2068
ECMO	5 (6.9)	0	5 (27.8)	0.0006
CRRT	8 (11.1)	0	8 (44.4)	<0.0001
Convalescent plasma transfusion	5 (6.9)	0	5 (27.8)	0.0006
Nasal catheter or face mask for oxygen therapy	20 (27.8)	20 (37.0)	–	NA
HFNC	16 (22.2)	16 (29.6)	–	NA
BiPAP ventilation	18 (25)	18 (33.3)	–	NA
Invasive mechanical ventilation	18 (25)	0	18 (100)	<0.0001
Days of hospital stays-median (IQR)	28 (19–38)	23 (16–35)	40 (31–49)	<0.0001
Outcome				
Shock	5 (6.9)	0	5 (27.8)	0.0006
Secondary infection	11 (15.3)	1 (1.9)	10 (55.6)	<0.0001
Discharged	69 (88.9)	54 (100)	15 (55.6)	0.0137
Died	3 (4.2)	0	3 (16.7)	0.0137

*Data are median (IQR, interquartile range) or n/N (%), where N is the total number of patients with available data. P-values comparing severe and critical groups are from χ^2^, the Fisher’s exact test, or Mann–Whitney U test. MP, methylprednisolone; ECMO, extracorporeal membrane oxygenation; CRRT, continuous renal replacement therapies; HFNC, high flow nasal cannula; BiPAP, bilevel positive airway pressure.*

### Clinical Characteristics

The age of all the patients was approximately 62 years. Common symptoms on admission were similar to those previously reported (Chen et al., 2020; Huang et al., 2020; Wang et al., 2020). Fever (86.1%) and cough (59.7%) were the most common symptoms at the early stage. Four cases were asymptomatic on admission. However, these patients developed dyspnea within 4 days and required oxygen therapy. Laboratory testing immediately on admission detected a significant increase in D-dimer, IL-6, and CHE (cholinesterase) in critical cases, which is consistent with previously reported risk factors in death (Zhou et al., 2020).

### All Patients Required Oxygen Therapy

Most patients (93.1%) had bilateral pneumonia on admission. Some of those in the severe and critical groups had PaO_2_/FiO_2_ >300 on admission, but, in the next week, their PaO_2_/FiO_2_ dropped to <300 or 100. Fifty-four patients in the severe group accepted non-invasive oxygen therapy. All eighteen patients in the critical group underwent invasive mechanical ventilation and ICU care.

### Use of Corticosteroids

We ordered a low-to-moderate dose of <0.8 mg/kg/methylprednisolone for treatment with a median duration of 5 days. The median cumulative dose of methylprednisolone was 240 mg per person. The median days from the onset to methylprednisolone use was 8 days (Table 5).

### Secondary Infection

There were no significant differences in secondary infections or antibiotic use between the corticosteroid-treated and corticosteroid-free groups (Table 1). In the severe group, only one patient’s sputum was cultured, detecting *Pseudomonas aeruginosa*; this patient was 62 years old and had diabetes. After antibiotic treatment, he was discharged on recovery. Due to invasive ventilation, secondary infections (positive culture of bacteria or fungi) were more common in critical cases, but no bacteria or fungi were observed by blood culture.

**FIGURE 1:**
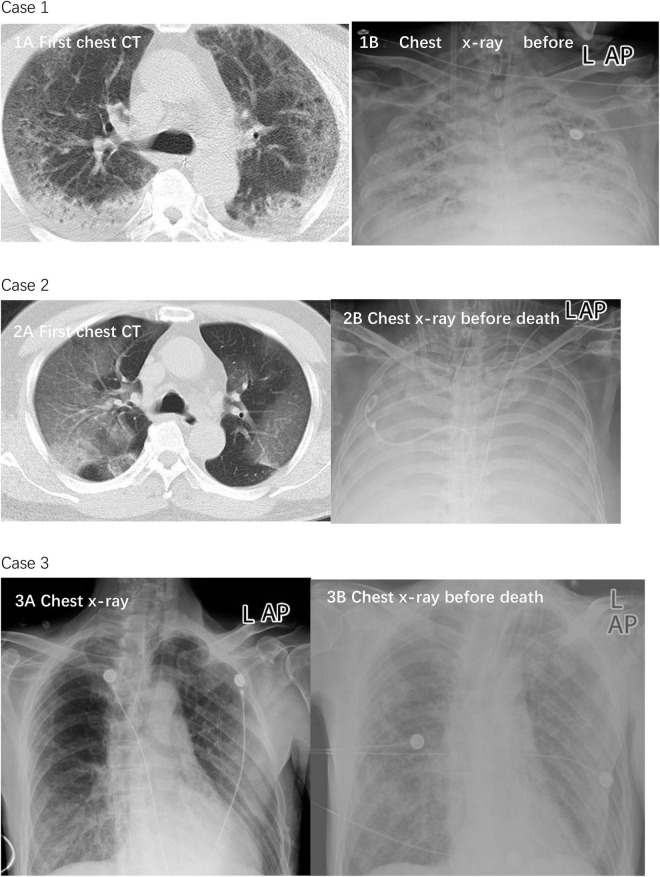
Chest CTs and chest x-rays of three died patients. Case 1: Chest CT was obtained on January 23 **(1A)**, 2020. The diffuse density of both lungs increased unevenly, and multiple patchy density shadows were observed in each lobe. Most of the lesions showed ground glass density and some consolidation, which was observed near the dorsal subpleural. The lesions were diffusely distributed in multiple small cystic low-density areas. Chest x-ray on February 16, 2020 showed worse status **(1B)**. The transmittance of both lungs is reduced. A diffuse flocculent high-density shadow is seen. The boundary is not clear. A bilateral diaphragmatic surface is blurred. Case 2: Chest CT was obtained on January 13, 2020 **(2A)**. The transmittance of both lungs is reduced. A diffuse flocculent high-density shadow is seen. The boundary is not clear. A bilateral diaphragmatic surface is blurred. Chest x-ray on February 16 showed worse status **(2B)**. Both lungs showed diffuse high-density shadows, with “white lung”-like changes. The bilateral diaphragmatic surface and costophrenic angle were unclear. Case 3: Chest x-ray was obtained on January 27, 2020 **(3A)**. Double lungs scattered in multiple patchy densities increased the shadow; the boundary is not clear, especially the double-lung lower field. The bilateral diaphragmatic surface is smooth; the bilateral costal diaphragmatic angle is sharp. Chest x-ray showed worse status on February 21 **(3B)**. In each field of both lungs, multiple patchy densities that increased shadows were seen, which were thicker than before, with an unclear boundary. The double costophrenic angle was slightly obtuse, with a small amount of pleural effusion on both sides.

As reported before (Guan et al., 2020; Ruan et al., 2020), the leading cause of mortality of the three cases was severe acute respiratory distress syndrome (ARDS). In all cases, imaging showed multiple mottling and ground-glass opacities (Figure 1).

Multivariable logistic regression analysis was performed to examine the independent effect of corticosteroid therapy on mortality with independent variables, including corticosteroid therapy and other treatment and laboratory results. No variables showed a significant influence on mortality (*p* > 0.05).

## Discussion

This was a single-center, retrospective study on the clinical characteristics and treatment of patients with severe or critical COVID-19. Although Oxford University carried out a randomized controlled clinical study on corticosteroid therapy for COVID-19 (RECOVERY trial), non-severe cases were included in their study, which made the cases not benefit and highlighted the double-edged sword of corticosteroid, and the final result proved that corticosteroid led to increased mortality of non-severe cases (Group et al., 2021). Since mild/moderate pneumonia was not considered for a long time ago in China, because we have come to a consensus that corticosteroid use here does more harm than good. This kind of clinical research like RECOVERY trial is not ethical if in China.

There is no evidence to support that LPV/r or other antiviral therapies can significantly reduce mortality (Jiang Hua et al., 2020). The symptoms of the fatal cases continued to worsen after later testing negative for the virus, and the three deaths were mainly caused by ARDS (Figure 1). This suggests that critical cases might be immunologically mediated; immune-modulatory therapies have been approved by many physicians (Shang et al., 2020; Wu et al., 2020a; Zhao et al., 2020).

The data in this study suggest that corticosteroid treatment for COVID-19 does not significantly increase mortality among patients with severe disease. All patients with invasive ventilation were treated with methylprednisolone, and BiPAP ventilation and HFNCO were more common in the corticosteroid group than in the corticosteroid-free group. Although cases were more severe in the corticosteroid treatment group, there was no significant increase in mortality. In general, a higher death rate accompanies more severe the disease. However, there was no increase in mortality in patients with more severe disease compared with those with relatively less severe disease. This suggests that corticosteroid treatment benefits severe COVID-19 cases. A retrospective Cox survival analysis conducted for severe cases at Wuhan Jinyintan Hospital (Wuhan, China) suggested that treatment with methylprednisolone is beneficial for patients with COVID-19, who develop ARDS (Wu et al., 2020a). But, in this study, multivariable logistic regression analysis suggested that methylprednisolone was not a significant influence on mortality. The reason for the different conclusion is due to selection bias that there were no corticosteroid-free cases in the critical group (invasive ventilation), and most patients with relatively mild were not treated with corticosteroid. The more severe the illness is, the more likely is a physician to choose corticosteroid therapy.

As of 30 April, 2020, 450 COVID-19 cases [wild-type virus (WT)] had been confirmed in Shenzhen, with three (0.6%) deaths to date. Compared to the report from the same period of 1,099 patients with COVID-19 by Zhong et al., the mortality rate in our study was significantly reduced in both severe cases [5.56% (3/54) vs. 8.1% (14/183)] and in those requiring invasive ventilation and glucocorticoids [16.6% (3/18) vs. 29.4% (5/17)] (Guan et al., 2020). Compared with other published clinical reports (Huang et al., 2020; Wang et al., 2020), the mortality rate of severe patients in Shenzhen was significantly lower. Different usage and dosage of corticosteroids may lead to different clinical outcomes. The patients in this study were treated with methylprednisolone at a low-to-moderate dosage (≤0.5–1 mg/kg/d; cumulative dosage, 240 mg) of short duration (5 days). No significant corticosteroid-related side effects occurred, such as secondary infections. However, in Russell et al. (2020), the dose was much higher and the duration longer than in our series. A prospective, randomized, double-blinded, placebo-controlled trial (Lee et al., 2004b) to assess the clinical efficacy of early corticosteroid (12 days of intravenous hydrocortisone 300 mg/d; or “Pulse” of intravenous high-dose methylprednisolone (500 mg/day for three consecutive days) up to a total of 3.0 g) use in patients with SARS suggested delayed clearance of viral RNA from the blood. In a case-control study of SARS-related psychosis (Lee et al., 2004a), 10,975-mg (the cumulative dose) hydrocortisone (equivalent to 2,195-mg methylprednisolone) was used, although the maximum cumulative dose in our study was only 480 mg. Apparently, reported complications associated with corticosteroid treatment, such as diabetes and avascular necrosis, were caused by a much higher dose or longer duration (Li et al., 2004; Xiao et al., 2004). As a double-edged sword, how to maintain the good effect of corticosteroid treatment for COVID-19 most likely depends on the appropriate usage plan.

Optimal timing of corticosteroid treatment may be important for physicians to consider. CD4+ and CD8+ cell counts in the corticosteroid treatment group were significantly lower than those in the corticosteroid-free group, although we could not find a cut-off value to guide the timing of corticosteroid use. In the report of risk factors in mortality analyzed by Cao’s team (Zhou et al., 2020), the time from the illness onset to corticosteroid treatment was 12–133 days; in our study, it was approximately 8 days. Hyperinflammation, which is characterized by fulminant and fatal hypercytokinemia with multiorgan failure, or the “cytokine storm syndrome,” is identified as an important mechanism for death in COVID-19 (Mehta et al., 2020). Therefore, corticosteroids may be recommended as an important immunomodulatory therapy. As Huang et al. reported, increases in over twenty-two kinds of cytokines have been observed, including interleukin (IL)-2, IL-7, granulocyte colony-stimulating factor (GSF), interferon-γ, inducible protein (IP)-10, monocyte chemoattractant protein (MCP)-1, macrophage inflammatory protein (MIP)1-α, and tumor necrosis factor (TNF)-α (Huang et al., 2020). In their study, the median time from transfer to a designated hospital to blood sample collection was 4 days (IQR, 2–5), with a number of days from first admission to transfer of five (IQR, 1–8). In our study, days from the illness onset to admission was 5, and we found significantly increased IL-6 on admission, which suggests that the cytokine storm syndrome is most likely to occur in the 1st week of the clinical course. However, thirteen (72%) critical patients in our study accepted invasive mechanical ventilation 12 days after the symptom onset. Therefore, early detection of the cytokine storm rather than relying only on clinical signs to determine corticosteroid intervention timing may lead to better outcomes, such as avoiding invasive ventilation (Shang et al., 2020; Zhao et al., 2020). More clinical studies are needed to confirm this hypothesis and the influence factors that initiate corticosteroid therapy.

Our study has some limitations: The sample size of this study is too small, and more severe cases in the control group (corticosteroid free) are needed, especially those with invasive ventilation. This may be related to the clinical experience of the research team in treating viral pneumonia for many years, such as H7N9 avian influenza virus pneumonia. We have rich experience in the use of corticosteroid; most doctors are willing to use low-to-median doses of corticosteroid in the treatment of severe COVID-19. In the future, we may summarize the efficacy and experience of corticosteroid in patients with viral pneumonia. This was a retrospective study, and the longer prognosis after discharge was not included in the observation, which was also one of the shortcomings of this study.

## Conclusion

Early use of a low-to-moderate dosage of short duration of corticosteroids may be accurate immune-modulatory treatment and offers benefits to patients with severe COVID-19 until the availability of proven antiviral therapy or vaccine administration.

## Data Availability Statement

The original contributions presented in this study are included in the article/supplementary material, further inquiries can be directed to the corresponding author/s.

## Ethics Statement

The studies involving human participants were reviewed and approved by the Institutional Ethics Board of Shenzhen Third People’s Hospital. Written informed consent for participation was not required for this study in accordance with the national legislation and the institutional requirements.

## Author Contributions

LY: full access to all of the data in the study, takes responsibility for the integrity of the data and accuracy of the data analysis, drafting of the manuscript, and statistical analysis. LY and YL: concept and design and obtained funding. LY, LP, WW, MC, CC, and FW: acquisition, analysis, or interpretation of data. JS: critical revision of the manuscript for important intellectual content. All authors contributed to the article and approved the submitted version.

## Conflict of Interest

The authors declare that the research was conducted in the absence of any commercial or financial relationships that could be construed as a potential conflict of interest.

## Publisher’s Note

All claims expressed in this article are solely those of the authors and do not necessarily represent those of their affiliated organizations, or those of the publisher, the editors and the reviewers. Any product that may be evaluated in this article, or claim that may be made by its manufacturer, is not guaranteed or endorsed by the publisher.
